# RNA sequencing data of Vemurafenib-resistant melanoma cells and parental cells

**DOI:** 10.1016/j.dib.2020.105610

**Published:** 2020-04-22

**Authors:** Kun Zhao, Yanrong Lu, Younan Chen, Jingqiu Cheng, Wengeng Zhang

**Affiliations:** a Key Laboratory of Transplant Engineering and Immunology, NHFPC; Regenerative Medicine Research Center, West China Hospital, Sichuan University, Chengdu, Sichuan, China; b Precision Medicine Key Laboratory of Sichuan Province and Precision Medicine Center, West China Hospital, Sichuan University, Chengdu, Sichuan, China

**Keywords:** Melanoma, BRAF, Vemurafenib, resistance, RNA-seq

## Abstract

Melanoma is a type of malignant tumor derived from melanocytes, most of which occur in the skin, and a few occur in the mucosa and choroid. BRAF mutations occur in approximately 50% of melanoma patients. Vemurafenib is a specific and potent BRAF inhibitor that significantly prolongs progression-free survival in patients with BRAF mutant melanoma. But most patients have tumor recurrence after 7-9 months. Drug resistance severely limits the long-term clinical effects of targeted drugs. To explore the mechanism of melanoma resistance to Vemurafenib, the transcripts of Vemurafenib-resistant melanoma A375R cells and the parental A375 cells were sequenced. For more insight please see Transcripts 202 and 205 of IL-6 confer resistance to Vemurafenib by reactivating the MAPK pathway in BRAF(V600E) mutant melanoma cells [1]. RNA-seq data has been uploaded to Sequence Read Archive (SRA), which allows researchers to obtain RNA sequence data for these cells.

Specifications table

**Table:** 

Subject	Oncology Science
Specific subject area	Tumor drug resistance, Transcriptomics
Type of data	Table Figure Transcriptome sequences (RNA-Seq clean reads)
How data were acquired	Images were captured using a Olympus IX70 microscope with camera model Olympus DP70 Sequencing data acquired by BGISEQ-500 sequencing platform Primers designed by Primer Premier 5 Quantitative real-time PCR was performed by a Chromo4 cycler
Data format	Raw Analyzed Filtered
Parameters for data collection	Images were captured in 200x (20x objective lens × 10x eyepiece) and 400x (40x objective lens × 10x eyepiece) magnifications. The RNA used for sequencing was obtained from Vemurafenib-resistant A375R and Vemurafenib-sensitive A375 melanoma cells.
Description of data collection	The RNA of A375 and A375R cells was sequenced by BGISEQ-500 sequencing platform in BGI tech, Shenzhen.
Data source location	Chengdu, Sichuan Province, China (N30°39′, E104°05′)
Data accessibility	Raw data (FASTQ) of Vemurafenib-resistant A375R and Vemurafenib-sensitive A375 melanoma cells have been deposited in NCBI Sequence Read Archive (SRA) database. Repository name: NCBI Sequence Read Archive (SRA) data base Data identification number: PRJNA602782 Direct URL to data: https://www.ncbi.nlm.nih.gov/bioproject/PRJNA602782
Related research article	Kun Zhao, Yanrong Lu, Younan Chen, Jingqiu Cheng, Wengeng Zhang, Transcripts 202 and 205 of IL-6 confer resistance to Vemurafenib by reactivating the MAPK pathway in BRAF(V600E) mutant melanoma cells, Experimental Cell Research. 2020 Mar 12:111942. doi:10.1016/j.yexcr.2020.111942.

## Value of the data

• BRAF inhibitor Vemurafenib can significantly prolong the progression free survival of melanoma patients with BRAF mutation, but drug resistance limits the clinical efficacy of Vemurafenib [2], [3], [4].
• Two sets of RNA sequencing data, Vemurafenib resistant A375R and Vemurafenib sensitive A375 melanoma cells, were reported to facilitate understanding of the resistance mechanism of melanoma.
• The analysis of differentially expressed genes between drug-resistant and sensitive cells is helpful to identify the key genes that contribute to drug resistance in melanoma cells.

## 1 Data Description

**Fig. 1:**
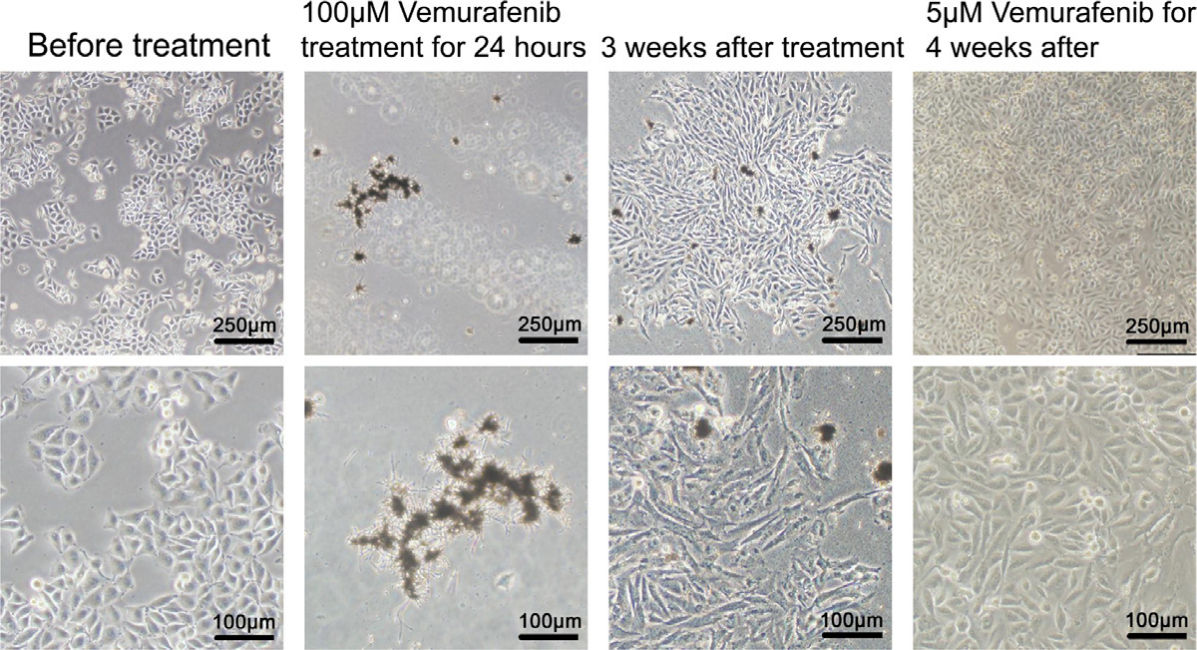
Morphology of human melanoma A375 cells. A375 cells were cultured in DMEM medium only (first column). After treated with 100 µM of Vemurafenib for 24 hours, most of the cells were dead and only a few cells were left alive (second column). Following the treatment, the cells were cultured in fresh DMEM without Vemurafenib for 3 weeks and cell colonies were formed (third column). Last column showed the cells from the colonies were cultured in DMEM with 5 µM of Vemurafenib for 4 more weeks.

Vemurafenib-resistant melanoma A375R cells were obtained by a high-dose and short-term drug treatment and then were maintained in DMEM cell culture medium with 5 µM of the drug. A375 cells were photographed before and after Vemurafenib treatment (Fig. 1). RNA-seq was performed on Vemurafenib-resistant A375R and Vemurafenib-sensitive A375 melanoma cells by BGISEQ-500 sequencing platform, 3 samples each group. Each sample produced 21.92 megabases of data on average and a total of 17475 genes were detected. After sequencing, all the original data were filtered to ensure data reliability. Data filtering includes removing the reads with low-quality, daptor sequences, contamination and high unknown base content. Clean reads were stored in NCBI Sequence Read Archive (SRA) data base with the bioproject number PRJNA602782. Quantitative real-time PCR (qPCR) was used to verify the differentially expressed genes between A375R and A375 cells found by RNA-seq. The primer sequence for the qPCR is shown in Table 1. The results showed that IL-6 was highly expressed in A375R cells and consisted of three transcripts, IL6-201, IL6-202 and IL6-205.

**Table 1:** Primer sequences for RNA-Seq validation.

Gene	Forward Primer (5’-3’)	Reverse Primer (5’-3’)
GAPDH	ACCACAGTCCATGCCATCAC	TCCACCACCCTGTTGCTGTA
IL-6	GAAAGCAGCAAAGAGGCACT	TTTCACCAGGCAAGTCTCCT
IL18	GCTGAAGATGATGAAAACCTGG	CAAATAGAGGCCGATTTCCTTG
IL36B	CGTGATTCTCGACAGATGGTGTGG	ACAGGCTTAATGCTGCGGCTAAG
ILDR2	TCGCATGGGAGAATCCTTGG	CTCGAACAGTCCTCCTGCTG
IL-32	ATGCACCAGGCCATAGAAAG	TCCAGGTAGCCCTCTTTGAA
TBC1D3D	GGCAAGAGGTCATCTGAGCACATC	CCGCTGCTTGGTTCCGTATCG
SPANXC	CCAACGAGGCCAACGAGATGATG	GGCGTGGTCATTCACCAGTTCC
COX7B2	GCTAGCCAGTGGAACTGCTT	TCTGCCAACAGGGGATAGGT
SSTR1	TGTTGTACACATTTCTCATGGG	CATCTTAGCAATGATGAGCACG
VCX3B	AGAGCGAGCTGGAGGAACCAC	CTCCACCTGGCTCTCCTGACTC
MYOCD	CGAAGTCCAACTGCTGTCCTCAC	GCTCATCACTGTCGGTGGCATAG
SPANXB1	AGTGTCCGCAGGCTGAAGAGG	GGTCTCCGGCATCGTCTTGTTG
SH3GL2	GGGCTGTGATGGAAATAATGAC	GTGTTGATCATGCTGAGCTTAG
OR51B5	GCTGATACCACCTTCAACCGACTG	GACACAGGTAATGAGAGCCTTGGC
BASP1	GGAAGCGCCTAGTTCCACAC	TTGGTCGGAATTAGCTGCCG

## 2 Experimental Design, Materials, and Methods

### 2.1 Cell Lines

A375, BRAF mutant human melanoma cell line, was obtained from the Shanghai Institutes for Biological Sciences (Shanghai, China) and was cultured in DMEM (HyClone, Logan, Utah, USA) supplemented with 10% FBS (Biological Industries, Israel) and 1% penicillin/streptomycin (Life Sciences, USA). All cell lines were tested by STR profiling (Feiouer, Chengdu, China). A375R cells were obtained from A375 cells treated with Vemurafenib (Selleckchem, Houston, TX, USA) and grown in DMEM supplemented with 10% FBS, streptomycin, penicillin, and 5 µM of Vemurafenib.

### 2.2 Isolation of total RNA and quantitative real-time PCR

Trizol isolation regent (life technologies, USA) was used to isolate RNA from A375 and A375R cells. Spectrophotometric analysis was used to guarantee high quality RNA for RNA-seq. SYBR Green PCR Master Mix Kit (Vazyme, Nanjing, China) and specific primers were used for quantitative real-time PCR.

### 2.3 cDNA library construction

Total RNA was processed by mRNA enrichment and rRNA removal. The obtained RNA was segmented with interrupt buffer and reverse transcribed into cDNA. The double-stranded cDNA was specifically modified and amplified by PCR with specific primers. The PCR product was heat denatured into a single strand, and then the single strand DNA library was obtained by cyclizing the single strand DNA with a bridge primer.

### 2.4 Sequencing and RNA-seq analysis

cDNA library was further sequenced using BGISEQ-500 platform in BGI tech. After sequencing, the raw data was filtered by Trimmomatic. The filtering process was mainly to remove the raw reads with adapters, the reads with more than 5% of unknown bases (N) and low-quality reads. The remaining cleans after filtering were called "clean cleans". Clean cleans were counted by the filtering software SOAPnuke and uploaded to NCBI Sequence Read Archive (SRA) database.

### 2.5 RNA-seq analysis

The original image data obtained by sequencing were transformed into the original sequence data (raw data or raw reads) by base calling, which were saved as FASTQ format files and contained the reads sequence and sequencing quality information. The quality control (QC) of the raw reads was then carried out to determine whether the sequencing data were suitable for subsequent analysis. After the QC was qualified, the filtered clean reads were compared with hg19 (GRCh37) reference human genome by HISAT software. After the comparison, sequence alignment quality control was determined by counting the comparison rate and the reads distribution on the reference sequence. Finally, a more in-depth analysis of sequencing data that complied with quality control standards was conducted, including the differential enrichment analysis of GO functions and the significant enrichment analysis of pathways among the differentially expressed genes.

## Appendix Supplementary materials

Image, application 1

## References

[1] Zhao K., Lu Y., Chen Y., Cheng J., Zhang W. (2020). Transcripts 202 and 205 of IL-6 confer resistance to Vemurafenib by reactivating the MAPK pathway in BRAF(V600E) mutant melanoma cells. Experimental cell research.

[2] Schadendorf D., van Akkooi A.C.J., Berking C., Griewank K.G., Gutzmer R., Hauschild A. (2018). Melanoma. The Lancet.

[3] Kim A., Cohen M.S. (2016). The discovery of vemurafenib for the treatment of BRAF-mutated metastatic melanoma. Expert opinion on drug discovery.

[4] Garbe C., Eigentler T.K. (2018). Vemurafenib. Recent results in cancer research. Fortschritte der Krebsforschung. Progres dans les recherches sur le cancer.

